# ISMAR-study presentation: in-hospital epidemiology and clinical management of respiratory and cardiac comorbidities in cardiac and respiratory disease units

**DOI:** 10.1186/2049-6958-9-28

**Published:** 2014-05-20

**Authors:** Roberto Tramarin, Mario Polverino, Maurizio Volterrani, Bruna Girardi, Claudio Chimini, Nicolino Ambrosino, Fernando De Benedetto, Cesare Proto

**Affiliations:** 1 Division of Cardiac Rehabilitation, IRCCS Policlinico San Donato - Care and Research Institute, Piazza Malan 1, I-20097 San Donato Milanese, Milan, Italy; 2 High Specialty Provincial Pulmonologic Unit, “Scarlato” Hospital, Scafati, (SA), Italy; 3 Division of Cardiac Rehabilitation, IRCCS San Raffaele Pisana - Care and Research Inst., Rome, Italy; 4 Division of Cardiology, Sant’Anna Hospital, Brescia, Italy; 5 Division of Pneumology, University Hospital Auxilium Vitae, Volterra, Italy; 6 Division of Pneumology, Ss.Annunziata Hospital, Chieti, Italy

**Keywords:** Comorbidity, Diagnostic procedure, Epidemiology, In-hospital, Management, Treatment

## Abstract

**Background:**

Cardiovascular and respiratory diseases are leading causes of morbidity and their co-occurrence has important implications in mortality and other outcomes. Even the most recent guidelines do not reliably address clinical, prognostic, and therapeutic concerns due to the overlap of respiratory and cardiac diseases.

**Study objectives and design:**

In order to evaluate in the reality of clinical practice the epidemiology and the reciprocal impact of cardio-pulmonary comorbidity on the clinical management, diagnostic workup and treatment, 1,500 cardiac and 1,500 respiratory inpatients, admitted in acute and rehabilitation units, will be enrolled in a multicenter, nationwide, prospective observational study.

For this purpose, each center will enroll at least 50 consecutive patients. At discharge, data analysis will be aimed at the definition of cardiac and pulmonary inpatient comorbidity prevalence, demographic characteristics, length of hospital stay, and risk factors, taking into account also procedures, pharmacological and non-pharmacological treatment, and follow up in patients with cardio-respiratory comorbidity.

**Conclusions:**

The purely observational design of the study aims to give new relevant information on the assessment and management of overlapping patients in real life clinical practice, and new insight for improvement and implementation of current guidelines on the management of individual diseases.

## Introduction

Cardiovascular diseases (and chronic heart failure as a common ending stage of most cardiac diseases) together with chronic obstructive pulmonary disease (COPD) are both well-known causes of comorbidity and mortality. According to the latest analysis of the World Health Organization (WHO), chronic diseases are the cause of mortality for more than 36 million people every year. Cardiovascular diseases account for most chronic diseases-deaths (17.3 million people annually) but also respiratory diseases, and specifically COPD, have a high epidemiological impact (4.2 million deaths annually), with a trend to represent, within a few years, the third absolute cause of mortality. WHO predictions show that these groups of chronic diseases will be responsible for a significantly increased total number of deaths in the next decade (with a global mortality increase of 15% between 2010 and 2020) [1].

Multimorbidity is very frequent in patients with chronic disease and it is almost the norm in subjects over 65 years; up to one third of heart failure (HF) patients is affected with COPD and vice versa [2]. The prevalence of COPD among individuals with HF ranges from 20% up to 32% of cases, and 10% of HF inpatients also suffer from COPD. On the other hand, prevalence of HF in COPD patients exceeds 20%. Even though these conditions have been generally studied separately, several reports have demonstrated that these conditions frequently coexist, with important prognostic and therapeutic implications.

However, despite both conditions share some risk factors including cigarette smoking, advanced age, and systemic inflammation, surprisingly there are only few prospective trials investigating the close interaction between these two diseases under an epidemiological and clinical point of view.

Furthermore, it is interesting to observe that issues related to clinical, prognostic, therapeutic, and care implications are not considered in depth in even the most recent respective guidelines due to their overlap.

Moreover, the advantages or the potential long-term risks of concomitant treatments according to the individual guidelines in COPD and HF patients are unclear and undefined in the overlap syndrome. The potential impact of beta-blockers in COPD patients, especially if some degree of asthma is associated, is well known: in fact in many countries bronco-obstruction remains a formal contraindication to beta-blockers prescription. Other drugs (including ACE-inhibitors and amiodarone) widely used in cardiac patients can adversely affect respiratory conditions; conversely many drugs useful and widely prescribed in COPD have frequent adverse cardiovascular effects. The interaction between the two different individual therapies could be critical in particular subgroups of patients as, for example, the elderly [3]. On the other hand, an integrated approach is mandatory, considering that beta-blocker therapy improves symptoms and survival in most heart diseases, but only 35% of patients with chronic HF and COPD receive beta-blockers, although it has been shown that beta1-selective blockade is safe in this group of patients [4].

It is also known that COPD, together with many other respiratory diseases, regardless of smoking history, represents an important risk factor for hospitalization and cardiovascular mortality. Some studies suggest that COPD and other respiratory conditions should be adequately taken into account and treated for their prognostic impact on many cardiac diseases [5]. Recently a *post hoc* analysis of the SHIFT study was conducted with the aim to evaluate the clinical profile of patients with congestive HF and COPD, the use of drugs in this population and the prognostic impact of these two comorbidities. COPD determined a worsening of the risk profile; beta-blockers were prescribed in 69% of COPD patients and in 92% of non-COPD ones, and the COPD – HF overlap resulted in a significantly worse prognosis [6].

Therefore, great interest has been shown towards a worthy search preview on treatment interactions between the two comorbidities, basically aimed at defining the pathophysiology of cardiac and pulmonary comorbidity and the determinant factors influencing the single disease progression in the presence of both. In this regard, recent prospective studies indicate a double protective role of ACE-inhibitors, angiotensin-receptor blockers and statins both on cardiac and pulmonary functions producing a reduction in hospitalization for cardiac events and in pulmonary exacerbations [7,8].

Thus, in view of these observations, we need that new prospective controlled trials are required in order to better understand the interconnection between cardiac and pulmonary diseases.

## Objectives of the study

The aim of the study is to obtain an updated snapshot of the cardiac and pulmonary comorbidity prevalence in a series of consecutive cardiac and pulmonary inpatients, by assessing the specific diagnostic workup and care profile. In the final stage an important objective will be the in-depth analysis of the concomitant treatment according to the two mutual comorbidities. For this purpose, clinical data referred to hospitalization will be acquired at patient discharge in order to create an adequate representative sample of current inpatient cardiac and respiratory comorbidities in real clinical practice. More in detail, data analysis will be in order to acquire cardiac and pulmonary inpatient comorbidity prevalence, demographic characteristics relative to cardiac and pulmonary comorbidity, length of hospital stay, evaluation of cardiovascular and pulmonary risk factors and other relevant comorbidities, and assessment of diagnostic procedures, pharmacological and non-pharmacological treatment*,* and follow up in patients with cardio-respiratory comorbidity.

## Study design and procedures

The ISMAR (**I**ndagine **S**ICOA-A**IMAR**) is a multicenter, prospective observational study in which the objective is to recruit 3,000 patients on discharge after hospital admission. The survey has been carried out in 28 SICOA (Società Italiana Cardiologia Ospedaliera Accreditata) network cardiology units and 28 AIMAR (Interdisciplinary Association for Research in Lung Disease) network pulmonary units, including intensive, invasive, heart failure and rehabilitation cardiac units, and intensive and not intensive, respiratory and rehabilitation pulmonary units, which were selected in order to produce a sample representative of the current national in-hospital care provision. A recruitment of at least 50 consecutive patients for each center is expected, in a period between December 2013 and March 2014. The study has been approved by the Ethical Committee IRCCS San Raffaele – Pisana, Rome, Italy on the 1^st^ July 2013 session, deliberated by PR n°23/13 on the 17^th^ July 2013. The data has been collected at the patient discharge/death on the basis of records within clinical documentation. In case of patients without pulmonary or cardiac comorbidity, data collection will be limited to the demographic and primary disease description; on the contrary for the patients with cardiac-pulmonary comorbidity, all data in the purpose of the study will be collected. Web-based electronic case report forms (e-CRF) will be used for data entry and transferred via web to SICOA Study Centre. e-CRF is designed according to a multiple choice style, with jump menus or select boxes in order to avoid the possibility of confounding answers. The following information will be collected via e-CRF: the definition of the cardiac or pulmonary in-hospital setting, length of stay, demographics, main admission cardiac or respiratory diagnosis and other relevant comorbidities, according to the IDC-9-CM 2007 diagnosis code system. All information has been collected through direct consultation of discharge records and clinical charts. Furthermore, information about cardiovascular and pulmonary risk factors, as well as on all diagnostic procedures during the hospitalization for patients with comorbidity, on pharmacological and not pharmacological treatments, and finally on post-discharge scheduled clinic and diagnostic procedures follow up has been collected (Table 1).

**Table 1:** Sections of the ISMAR electronic case report form

- Discharge unit classification	
- Hospital setting	
- In-hospital multidisciplinary facilities	
- Principal diagnosis (ICD-9-CM)	
- Respiratory/cardiac comorbidities	
- Other comorbidities	
- Functional variables	
	- *NYHA Class,*
	- *GOLD stage*
	- *HR*
	- *LVEF*
- Risk factors	
	- *smoking habits*
	- *high blood pressure*
	- *cholesterol*
	- *diabetes*
	- *chronic kidney disease*
	- *BMI*
	- *sedentary*
	- *depression*
	- *cognitive impairment*
- Diagnostic procedures	
- Therapy at discharge	
- Non-pharmacologic treatments at discharge	
- Hospital length of stay	
- Follow up program	

## Discussion

ISMAR is a prospective, multicenter, national-based observational study designed to provide specific information with regards to pulmonary and cardiac comorbidity. In this survey we will observe a series of consecutive patients constituting a representative sample of cardiac and pulmonary Italian adult inpatients observed by focusing on the influence of each morbidity on the other. The only exclusion criterion is a discharge diagnosis not relevant with the admission unit. This trial will provide updated and detailed information about these two comorbidities in terms of prevalence, influence of close interaction on hospital length of stay, performed and scheduled diagnostic procedures, care burden, pharmacological and non-pharmacological therapy.

Several reports have demonstrated that pulmonary and cardiac pathologies frequently coexist, with important prognostic and therapeutic implications. Although the adherence to guidelines has been shown to be associated with improved outcomes, the implementation of these guidelines remains sub-optimal. Surveys and registries are effective tools for the assessment of guidelines implementation. ISMAR wants to yield an updated picture of the real in-hospital epidemiologic and clinical interconnection between pulmonary and cardiac comorbidity, and its impact on diagnostic and therapeutic strategies.

## Conclusions

The observational nature of this study is the clinical and management premise for future broad prospective trials in order to provide a comprehensive program to improve the clinical management of respiratory and cardiac comorbidities.

## Appendix

### ISMAR organization

Scientific Board

Enrico Pusineri (SICOA President), Cesare Proto (SICOA Past President), Fernando De Benedetto (AIMAR President), Nicolino Ambrosino (Volterra), Claudio Chimini (Brescia), Mario Polverino (Scafati), Roberto Tramarin (San Donato Milanese), Maurizio Volterrani (Roma)

### Data and e-CRF management

Centro Studi SICOA (Roma): Margot Bastianutti

App3 S.r.l. (Brescia): Carlo Rossini

### Statistical analysis

Centro Studi SICOA (Roma): Stefano Bonass, Rossella Moroni

### ISMAR Centers and Investigators Cardiac units

– Gian Mauro Mazzucco, Casa di Cura Villa Serena (Piossasco, TO, Piemonte)

– Massimo Piccinini, Casa di Cura Salus (Alessandria, Piemonte)

– Claudio Anzà, IRCCS Ospedale Multimedica (Castellanza, VA, Lombardia)

– Claudio Chimini, Istituto Clinico Sant’Anna (Brescia, Lombardia)

– Silvia Marangoni, Istituto Clinico San Rocco (Ome, BS, Lombardia)

– Alessandro Proto, Spedali Civili (Brescia, Lombardia)

–Cesare Proto, Istituto Clinico Sant’Anna (Brescia, Lombardia)

– Enrico Pusineri, IRCCS Policlinico San Donato (1.) (San Donato Milanese, MI, Lombardia)

– Cesare Storti, Casa di Cura Città di Pavia (Pavia, Lombardia)

– Roberto Tramarin, IRCCS Policlinico San Donato (2.) (San Donato Milanese, MI, Lombardia)

– Enrico Vizzardi, Università degli Studi di Brescia (Brescia, Lombardia)

– Paolo Barioli, Casa di Cura Rizzola (San Donà di Piave, VE, Veneto)

– Stefania De Feo, Casa di Cura Pederzoli (Peschiera del Garda, VR, Veneto)

– Fabrizio Proietti, Aurelia Hospital (Roma, Lazio)

– Stefano Rapino, Casa di Cura Villa Tiberia (Roma, Lazio)

– Maurizio Volterrani, IRCCS San Raffaele Pisana (Roma, Lazio)

– Francesco Caiazza, Casa di Cura Trusso (Ottaviano, NA, Campania)

– Pasquale Guarini, Clinica Villa dei Fiori (Acerra, NA. Campania)

– Bruno Ricciardelli, Clinica Mediterranea (Napoli, Campania)

– Lucia Urso, Casa di Cura Prof. Petrucciani (Lecce, Puglia)

– Pasquale Alcamo, Casa di Cura Triolo (Palermo, Sicilia)

– Bruno Aloisi, Centro Cuore Morgagni (Pedara, CT, Sicilia)

– Giuseppe Greco, Clinica del Mediterraneo (Ragusa, Sicilia)

– Nidan Torkmani, Casa di Cura Villa l’Ulivo-Carmide (Catania, Sicilia)

Respiratory Units:

– Delfino Legnani, Ospedale Luigi Sacco (Milano, Lombardia)

– Sergio Conte, Ospedale Vittorio Veneto (Vittorio Veneto, TV, Veneto)

– Emilio Marangio, Azienda Ospedaliero -Universitaria di Parma (Parma, Emilia Romagna)

– Francesco Gigliotti, IRCCS S. Maria agli Ulivi Fondazione Don Gnocchi (Pozzolatico, FI, Toscana)

– Rigoletta Vincenti, Azienda Ospedale USL 6 di Livorno (Livorno, Toscana)

– Stefano Carlone, AO San Giovanni – Addolorata (Roma, Lazio)

– Stefano Formisano, Fondazione Padre Alberto Mileno Onlus (Vasto Chiesti, CH, Abruzzo)

– Antonio Alfieri, Ospedale Umberto I di Nocera Inferiore (Nocera, NA, Campania)

– Francesco De Blasio, Casa di Cura Clinic Center S.p.A. (Napoli, Campania)

– Giuseppe De Simone, Casa di Cura Privata Villa Margherita (Benevento, Campania)

– Mario Del Donno, A.O. G. Rummo (Benevento, Campania)

– Mario Polverino, Ospedale Scarlato (Scafati, SA, Campania)

– Alessandro Sanduzzi Zamparelli, AOU Federico II (Napoli, Campania)

– Carlo Santoriello, Ospedale L. Curto di Polla (Polla, SA, Campania)

– Mauro Carone, Istituto Scientifico di Riabilitazione - Fondazione S. Maugeri (Cassano Murge, BA, Puglia)

– Paride Morlino, Ospedale S. Severo (San Severo, FG, Puglia)

– Eugenio Sabato, Ospedale “Melli” (San Pietro Vernotico, BR, Puglia)

– Filippo Andò, A.O.U. Policlinico G. Martino (Messina, Sicilia)

– NunzioCrimi, Ospedale Policlinico di Catania (Catania, Sicilia)

– Giovanni Passalacqua, Azienda Ospedaliera Ospedali Riuniti Papardo - Piemonte (Messina, Sicilia)

– Pietro Pirina, Clinica Pneumotisiologica - AOU Sassari (Sassari, Sardegna)

### Organization

The Italian Society of Accredited Hospitals Cardiologists (SICOA) and the Interdisciplinary Association for Research in Lung Disease (AIMAR) have the full responsibility of the ISMAR study, not only for the design and formulation, but also for the overall conduct of the study, including data monitoring and utilization of the results. Administrative location: SICOA – MICOM srl, Via Bernardino Verro 12, 20141 Milano, Italy.

## Competing interests

The authors declare that they have no competing interests.

## References

[B1] World Trade Organization The top 10 causes of death http://www.WHO.int/mediacentre/factsheets/fs310/en/

[B2] Le Jemtel TH Padeletti M Jelic S Diagnostic and therapeutic challenges in patients with coexistent chronic obstructive pulmonary disease and chronic heart failure J Am Coll Cardiol 2007 49 2 171 180 10.1016/j.jacc.2006.08.046 17222727

[B3] Boyd CM Darer J Boult C Fried LP Boult L Wu AW Clinical practice guidelines and quality of care for older patients with multiple comorbid diseases: implications for pay for performance JAMA 2005 294 716 724 10.1001/jama.294.6.716 16091574

[B4] Stefan MS Rothberg MB Priya A Pekow PS Au DH Lindenauer PK Association between β-blocker therapy and outcomes in patients hospitalized with acute exacerbations of chronic obstructive lung disease with underlying ischaemic heart disease, heart failure or hypertension Thorax 2012 67 11 977 984 10.1136/thoraxjnl-2012-201945 22941975 PMC4454610

[B5] De Miguel DJ Chancafe Morgan J Jiménez García R The association between COPD and heart failure risk: a review Int J Chron Obstruct Pulmon Dis 2013 8 305 312 23847414 10.2147/COPD.S31236 PMC3700784

[B6] Tavazzi L Swedberg K Komajda M Böhm M Borer JS Lainscak M Robertson M Ford I on behalf of the SHIFT Investigators Clinical profiles and outcomes in patients with chronic heart failure and chronic obstructive pulmonary disease: an efficacy and safety analysis of SHIFT study Int J Cardiol 2013 170 182 188 10.1016/j.ijcard.2013.10.068 24225201

[B7] Mancini GB Etminan M Zhang B Levesque LE FitzGerald JM Brophy GM Reduction of morbidity and mortality by statins, angiotensin-converting enzyme inhibitors, and angiotensin receptor blockers in patients with chronic obstructive pulmonary disease J Am Coll Cardiol 2006 47 12 2554 2560 10.1016/j.jacc.2006.04.039 16781387

[B8] Mortensen EM Copeland LA Pugh MJ Restrepo MI De Molina RM Nakashima B Anzueto A Impact of statins and ACE inhibitors on mortality after COPD exacerbations Respir Res 2009 10 45 10.1186/1465-9921-10-45 19493329 PMC2697974

